# Assessment of bony changes in temporomandibular joint in patients using cone beam computed tomography – a cross sectional study

**DOI:** 10.1186/s13005-023-00392-z

**Published:** 2023-10-28

**Authors:** Zahra Vasegh, Yaser Safi, Maryam Sanaei azar, Mitra Ghazizadeh Ahsaie, S. Marjan Arianezhad

**Affiliations:** 1 Department of Oral and Maxillofacial Radiology, School of Dentistry, Shahid Beheshti University of Medical Sciences, Tehran, Iran; 2 DDS, School of Dentistry, Shahid Beheshti University of Medical Sciences, Tehran, Iran; 3 Resident of Oral and Maxillofacial Radiology, Department of Oral and Maxillofacial Radiology, School of Dentistry, Shahid Beheshti University of Medical Sciences, Daneshju Blv, Velenjak St, Tehran, Iran

## Abstract

**Background and aim:**

The aim of this study is to evaluate the changes in the temporomandibular joint (TMJ) in patients with temporomandibular disorder (TMD) and the relationship between age, sex, and types of TMJ change using Cone Beam Computed Tomography (CBCT).

**Methods and material:**

CBCT records of 200 patients (123 women and 67 men) were retrieved and assessed. Right and left TMJs were evaluated separately, resulting in a total of 400 TMJs. The images were analyzed using On demand 3D Application The radiographic findings were classified as erosion, proliferative changes mainly, including flattening and osteophytes of the condyle, sclerosis, Ely cyst, hypoplasia and hyperplasia of the condyles, ankylosis, and joint cavity. Data analysis was performed using descriptive statistics, paired T-tests, and repeated measure ANOVA (Analysis of Variance) in SPSS Software.

**Results:**

The most prevalent types of condylar bony changes observed was osteophyte (63.5%) followed by flattening of the articular surface (42%), erosion (40%), ankylosis (10%) and sclerosis (10%). 7.5% of joints showed hyperplastic condyles but only 2% showed hypoplasia. The least prevalent change observed was Ely Cyst (1%). Osteophyte was the most prevalent change observed in all age groups and both sexes except for men aged 31 ~ 50, where flattening was more frequent. A statistically significant difference was found between sex and prevalence of erosion in the age group of 10 ~ 30 (*P* = 0.001); as well as between sex and condylar hyperplasia in the same age group.

**Conclusion:**

Based on the findings of this research, the prevalence of bony changes of TMJ from highest to lowest is as follows: osteophyte, flattening of the articular surface, erosion, ankylosis, sclerosis, hyperplastic condyles, hypoplastic condyles and Ely Cyst. CBCT is an accurate 3 dimensional imaging modality for assessment of TMJ bony structures.

## Introduction

The temporomandibular articulation is composed of bilateral, diarthrodial, temporomandibular joints (TMJs) [1]. It is formed by the glenoid fossa of the temporal bone and the mandibular condyle, making it one of the most complex joints in the human body [2]; The temporomandibular joint and its associated structures plays a crucial role in guiding mandibular motion and distributing stresses caused by daily activities such as chewing, swallowing, and speaking [3]. It is essential to comprehend how this structure develops and works to conduct accurate radiographic assessments.

Temporomandibular disorders refer to a group of disorders characterized by pain and tenderness in the TMJ, or the masticatory muscles, with limitation or deviated mouth opening and clicking sounds heard in the TMJ during mandibular function with pain in adjacent structures, and these do not interfere with growth or development and are not related to systemic diseases [4]. TMDs are associated with morphological and functional deformities and are the main source of orofacial pain of non-dental origin [5]. Degenerative bone changes involving the bone structures of the temporomandibular joint (TMJ) such as flattening, erosion, osteophytes, subchondral bone sclerosis and Ely cyst are commonly associated with TMDs.

The advent of CBCT has revolutionized the practice of dentistry [6], and CBCT is now considered the gold standard for imaging the oral and maxillofacial area [7] due to its numerous advantages, including reductions in exposure time, radiation dose, and cost in comparison to other imaging modalities [6]. CBCT has proven to provide reliable, precise, and clinically relevant TMJ data. As of now, CBCT has been found to be most useful in the evaluation of bony changes of the TMJ, such as fractures, ankylosis, dislocation, growth abnormalities, and various degenerative joint diseases including osteophytes, erosions, flattening, subchondral sclerosis, and pseudocysts [8]. CBCT provides 3D images of the mandibular condyle and surrounding structures to facilitate the analysis and diagnosis of bone morphological features, joint space, and the dynamic function, which serve as the critical keys to treatment outcome in patients with signs and symptoms of TMD [9, 10].

Currently, studies on bony changes in TMD patients using CBCT are still limited. The prevalence of degenerative changes in the TMJ differs across various authors and studied populations. To our knowledge, there is a lack of consensus in the literature regarding the impact of sex and age on the bony structures of the TMJ. As a result, this study aimed to evaluate the changes in the temporomandibular area of the jaw in patients with TMD and the relationship between age, sex, and types of TMJ change using CBCT.

## Materials and methods

This study was approved by the Research Deputy of shahid Beheshti University of Medical Sciences regarding ethical and methodological issues (code no: IR.SBMU.RIDS.REC.1394.156).

### Sample size

CBCT images of the TMJs of patients who attended a dental Radiology service in Shahid Beheshti University of Medical Science, Tehran, Iran from 2019 to 2021 were retrieved from the computer database and assessed. The sample included TMJ images of 200 patients (123 women and 67men). Right and left TMJs were evaluated separately, resulting in a total of 400 TMJs. Selection was a non-random sampling; calculated according to the formula:

$$\mathrm N={\mathrm z}_2\times\mathrm p\times(1-\mathrm p)/\mathrm d^2=1.96^2\times0.4\times0.6/0.07^2=200$$

Inclusion criteria were as follows:

1. Patients with a complaint of chronic temporomandibular joint pain.
2. Both condyles, joint cavity, and articular eminence should be in the field
3. Closed mouth CBCT images

Exclusion criteria were as follows:

1. Previous history of radiation treatment to the head and neck;
2. Previous history of TMJ surgery;
3. Congenital anomalies including TMJ
4. Previous history of trauma to jaw;
5. Pregnancy
6. Patients below 15 year of age
7. Image with poor quality due to patient movement and artifacts
8. Images with inadequate FOV

### Exposure parameters

The TMJ images of patients were obtained using a Soredex Scanora 3D 7.5 × 14.5cm CBCT unit (Helsinki;Finland) operating at 90 kVp, 8 mA with a voxel size of 0.25 mm.

### Measurements

The primary reconstruction of the raw data was restricted to the TMJ region. The thickness of the image slices was 1mm and the distance between slices was 1 mm [11]. For interpretation, real time reconstruction was performed using On Demand 3D Application; Version 10.0.1 and the axial, coronal, and sagittal two dimensional (2D) multi-planar reformatted slices were provided. Tomographic sections were taken in the curved planar reformation (panorex), a series of multiplanar reconstructions (cross-sections) and oblique planar reformation. Slides were prepared so that sagittal images were perpendicular to the condylar longitudinal axis, and coronal images were parallel to this axis [12, 13].

All images were examined by two experienced oral and maxillofacial radiologists independently to assess the position and morphology of the condyle. All images were viewed on the same monitor under the same conditions. In addition, all stages of image examination were performed totally blind. Two observers assessed these images twice with an interval of one week. To verify the reliability of measurements, the intra-observer agreement was calculated, using the Intra-class coefficient (ICC) test. The observers could change contrast and brightness of the images. In addition, there were no time limit for examinations.

The temporomandibular joints were assessed as follows:

0.  Absence of the subtype or variable.
1. The subtype or variable is located on the right.
2. The subtype or variable is located on the left.
3. The subtype or variable is located bilaterally.

The radiographic findings were classified as erosion, proliferative changes mainly, including flattening and osteophytes of the condyle, sclerosis, Ely cyst, hypoplasia and hyperplasia of the condyles,ankylosis, and joint cavity [14, 15].

Demographic information including age and gender were also recorded for each case. Hyperplasia and hypoplasia was diagnosed by asymmetry of the mandible, deviation of the mandible toward the unaffected size [16].

**Fig. 1:**
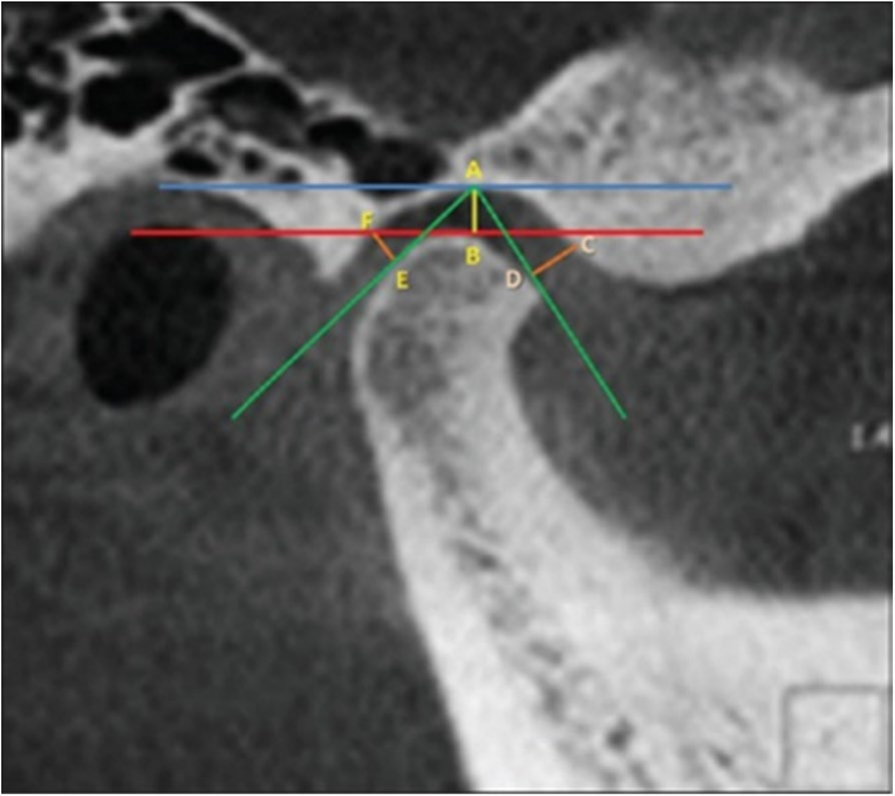
Measurement of anterior joint space, posterior joint space, and superior joint space (from sagittal view)

The study utilizes standardized linear measurements of space between the condyle and the articular fossa, following the method of Ikeda and Kawamura. From reconstructed sagittal sections, two horizontal lines were drawn; the first one is tangent to the uppermost area of the glenoid fossa (A) and parallel to Frankfort horizontal plane. The second line was drawn tangent to the most superior surface of the condyle (B). Two other lines were drawn tangential to the most anterior surface (D) and to the most posterior surface (E) of the condyle. A perpendicular distance between A and B, C and D, and E and F were then measured and considered as superior joint space (SJS), anterior joint space, and posterior joint space (PJS) distances, respectively (Fig. 1) [17].

In order to avoid misinterpretation, bone changes had to be found in at least two consecutive slices [18].

The criteria for the types of condylar change are as follows [11, 19]:

- Erosion: an area of decreased density or discontinuity or irregularity of the cortical bone
- Flattening: a flat bony contour deviating from the convex form
- Osteophytes: marginal bony outgrowths on the condyle
- Sclerosis: an area of increased density of cortical bone extending into the bone marrow
- Ely Cyst: or Pseudocysts are well-circumscribed osteolytic adjacent subcortical bone area without cortical destruction
- Condylar hypoplasia: defective formation of the condylar process that can be congenital or acquired
- Condylar hyperplasia: increased development of one mandibular condyle
- Ankylosis: fibrous or bony fusion between the condyle and fossa

**Fig. 2:**
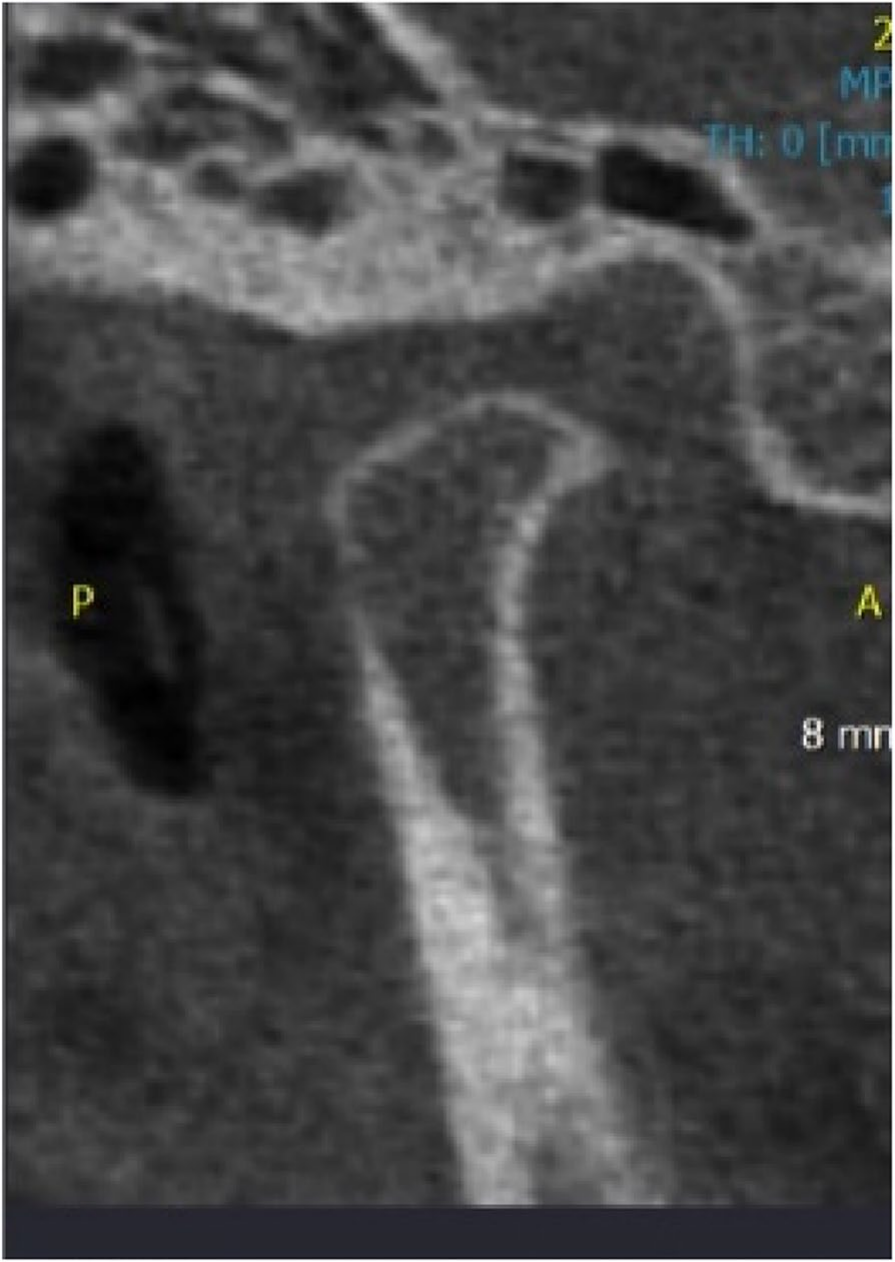
Sagittal CBCT view of right condyle showing present of osteophyte in anterior border of condyle

**Fig. 3:**
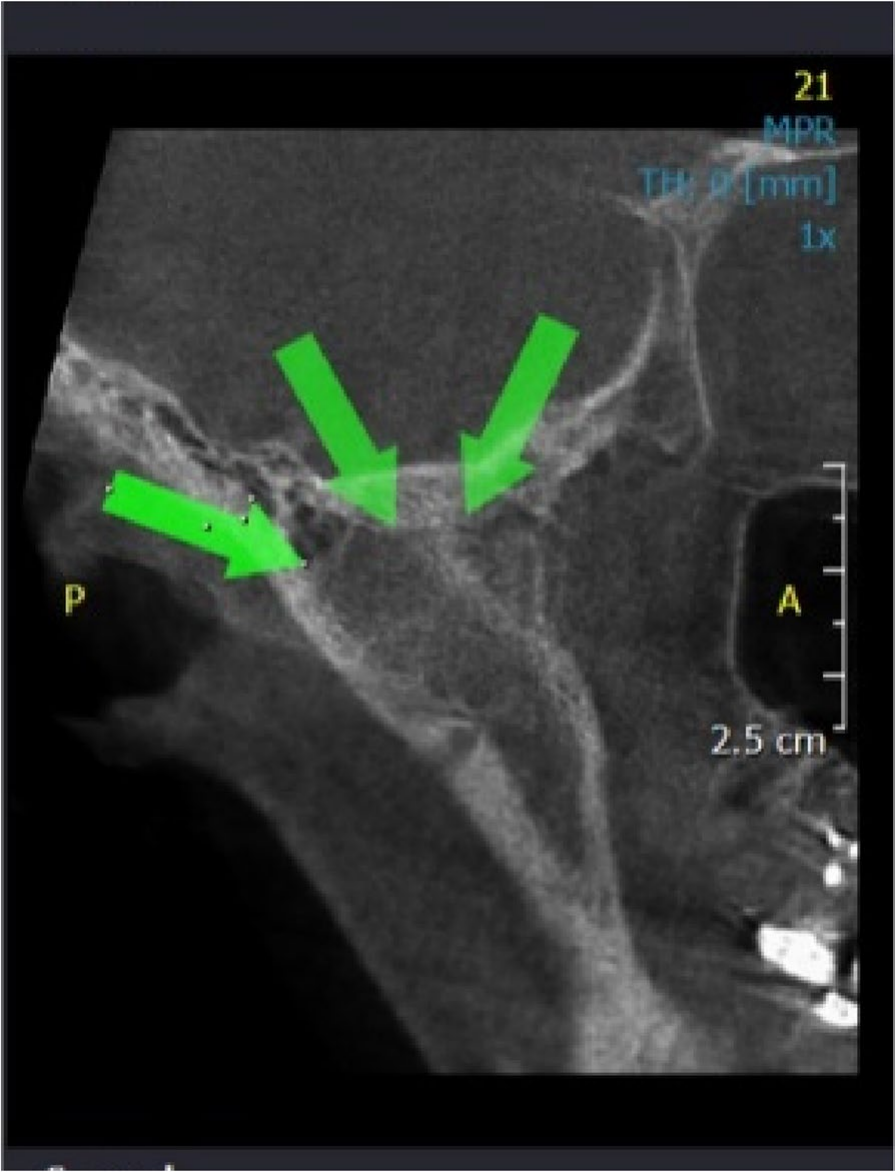
Coronal CBCT view showing condylar Ankylosis

**Fig. 4:**
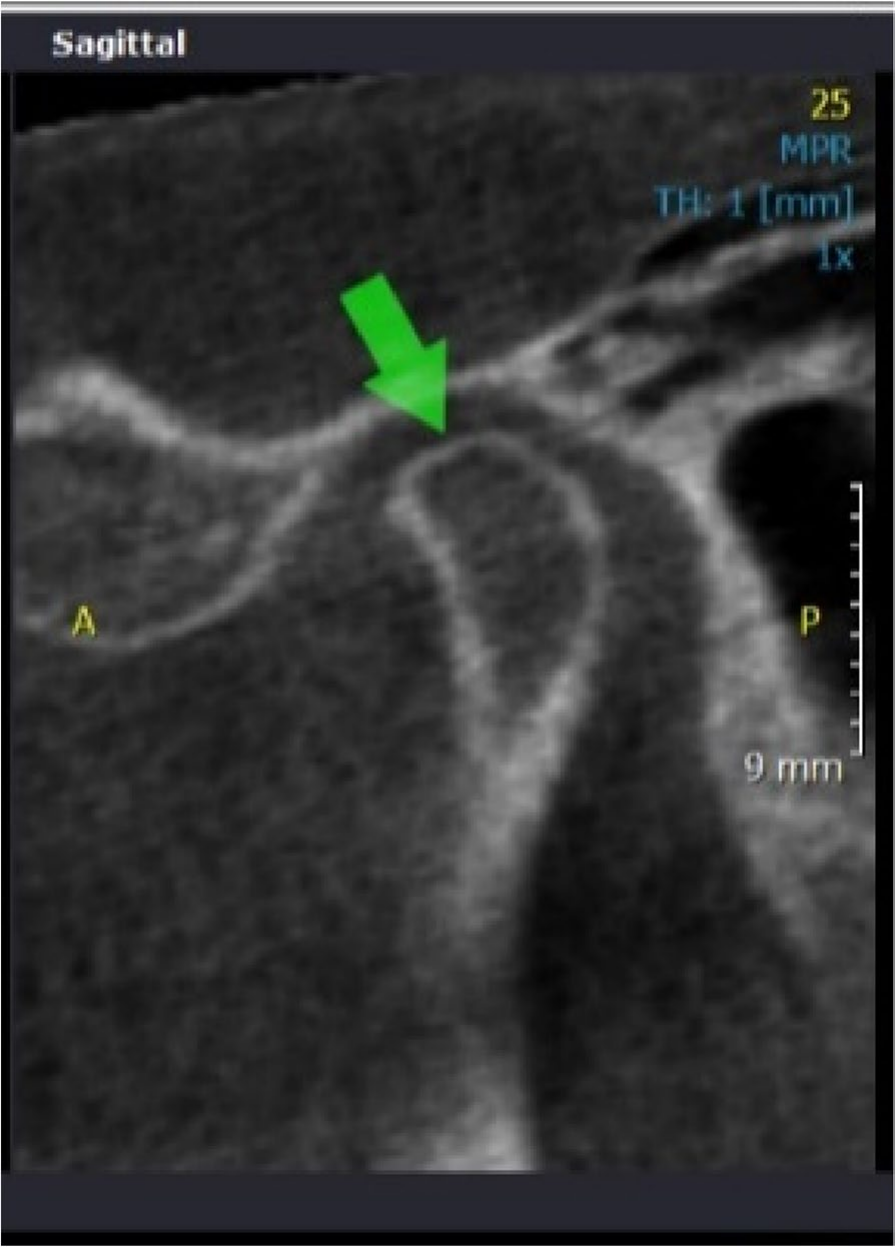
Sagittal CBCT view showing flattening on superior border of left condyle

**Fig. 5:**
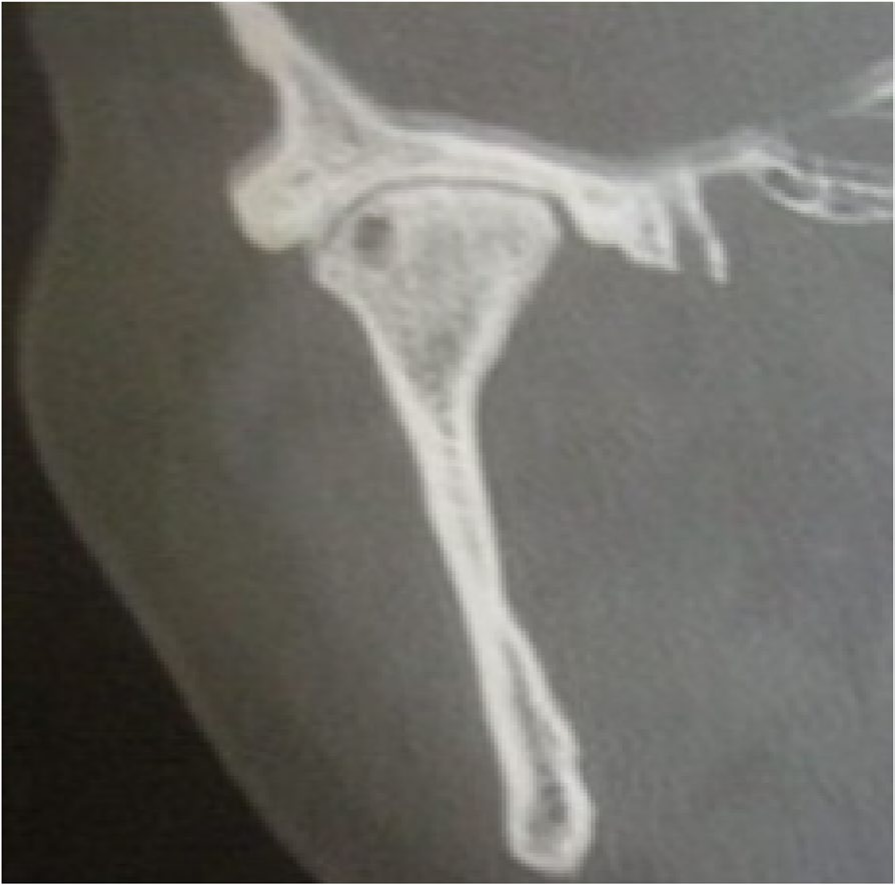
Coronal CBCT view showing Ely cyst in lateral pole of right condyle

**Fig. 6:**
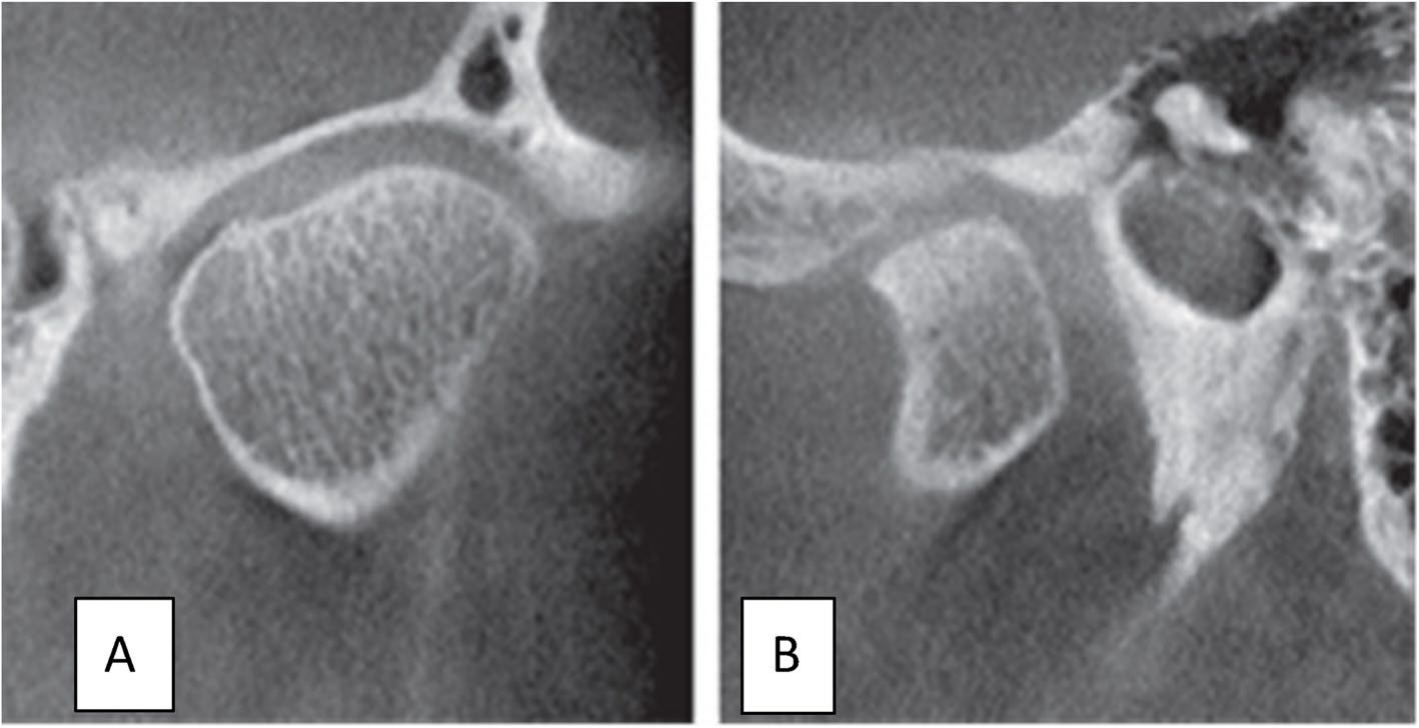
Coronal (**A**) and Sagittal (**B**) CBCT view showing Sclerosis

**Fig. 7:**
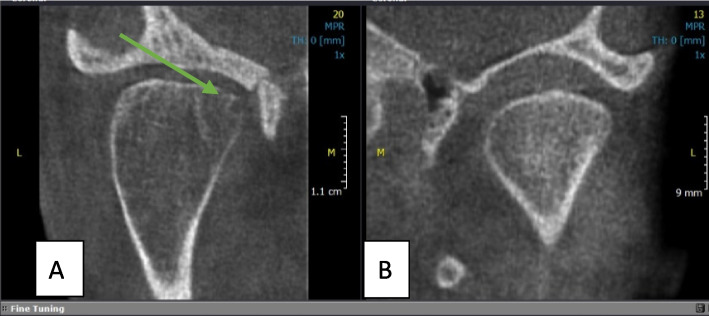
Coronal CBCT view of two patients with Condylar Hperplasia (**A** and **B**). Note the erosion in the medial pole of the Condyle (arrow in **A**)

Different bony changes seen by the two observers in DVT are shown in Figs. 2, 3, 4, 5, 6 and 7.

### Statistical analysis

All data were entered into a database system and evaluated using SPSS® for Windows version 21(SPSS Inc., Chicago, IL, USA, 2012). Patients' data were analyzed anonymously, with each case assigned a unique registration number to allow for explicit and anonymous identification. Data analysis was performed using descriptive statistics, paired T-tests, and repeated measure ANOVA (Analysis of Variance). Differences between the left and right joints of the patients were analyzed using the Pearson correlation test. The prevalence of 9subtypes among men and women was statistically analyzed using the chi-square test. The correlation between age and the observed radiographic findings was statistically analyzed with an independent T-test. The level of significance was set at *p* = 0.05.

## Results

### Intra-operator reliability

Measures for the first and second replicates of 20 patients were recorded and intra-class correlation coefficients (ICC) were established for all measurements. Most measures demonstrated a high degree of reliability between the first and second replicates with ICC values exceeding from 0.80 to 0.85.

### Demographic assessments

**Fig. 8:**
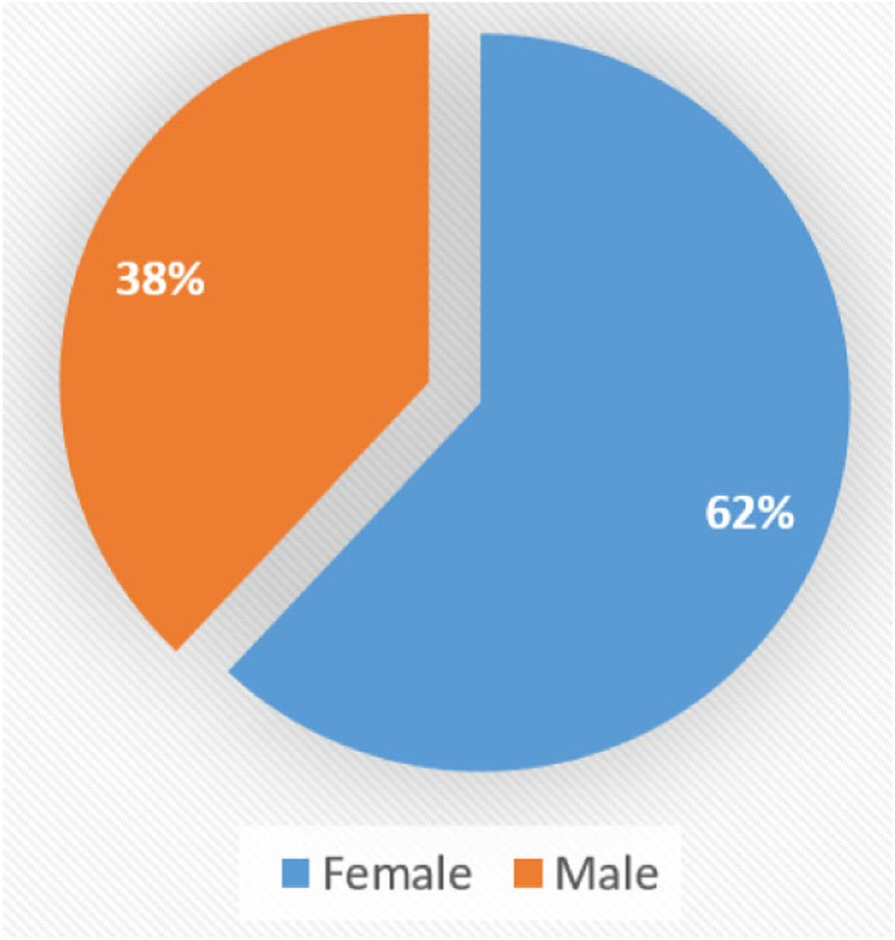
Distribution of gender

In this study, CBCT images of 200 patients (123 women and 76 men) resulting in a total of 400 TMJs; were assessed: as shown in Fig. 8. Subject age ranged from 11 to 70 years; divided in 3groups: (10–30) – (31–50) – (51–70) (Table 1).

**Table 1:** Distribution of age

Distribution of age	Number
10–30	93 (46.5%)
31–50	75 (37.5%)
51–70	32 (16%)

**Fig. 9:**
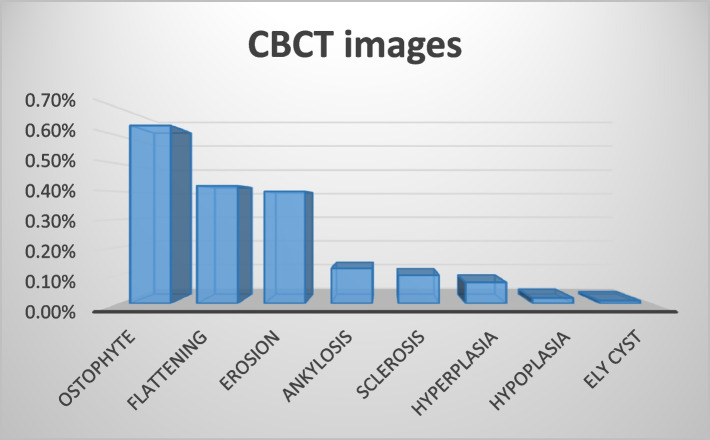
Prevalence of Bony changes of TMJ

The most prevalent types of condylar bony changes observed was osteophyte (63.5%; *N* = 254) followed by flattening of the articular surface (*N* = 168; 42%;), erosion (*N* = 160; 40%), ankylosis (*N* = 40;10%) and sclerosis (*N* = 40;10%). 7.5%(*N* = 30) of joints showed hyperplastic condyles but only 2% (*N* = 8) showed hypoplasia. The least prevalent change observed was Ely Cyst (*N* = 4;1%) (Fig. 9).

Table 2 shows prevalence of TMJ disorders according to the age group and sex. Osteophyte was the most prevalent change observed in all age groups and both sexes except for men aged 31 ~ 50, where flattening was more frequent) *N* = 16;59.30%;). In addition, in men of 51 ~ 70 Erosion and flattening were both equally the most prevalent change(*N* = 9; 60%). In the age of 10 ~ 30, a statistically significant difference was found between male and female participants in the prevalence of erosion (*P* = 0.001). Additionally, there was a marginal difference between male and female participants in the prevalence of condylar hyperplasia in the same age group (*P* = 0.052).

**Table 2:** Prevalence of TMD disorders for all participants by age groups and sex

Age range	Sex	Osteophyte	Erosion	Flattening	Sclerosis	Ely cyst	State-Condylar			Ankylosis
							Normal	Hypo	Hyper	
10–30	Male	24	21	9	3	0	32	2	0	5
	*N* = 34	-70.60%	-61.80%	-26.50%	-8.80%		-94.10%	-5.90%		-14.70%
	Female *N* = 59	38	14	24	9	0	48	2	9	8
		-64.40%	-23.70%	-40.7	-15.30%		-81.40%	-3.40%	-15.30%	-13.60%
	*P* value	0.54	0.001	0.17	0.52		0.052			1
31–50	Male *N* = 27	14	9	16 (59.30%)	4	0	25	0	2	3 (11.10%)
		-51.90%	-33.30%		-14.80%		-92.60%		-7.40%	
	Female *N* = 48	32 (66.7%)	21	24	4	0	44 (91.70%)	0	4	7 (14.60%)
			-43.80%	-50%	-8.30%				-8.30%	
	*P* value	0.206	0.38	0.44	0.45		1			0.74
51–70	Male *N* = 15	9	9	5	0	0	15 (100%)	0	0	2 (13.30%)
		-60%	-60%	-33.30%						
	Female	9	5	5	0	2	16	0	0	0
	*N* = 16	-56.30%	-31.30%	-31.30%		-12.50%	-100%			
	*P* value	0.83	0.11	1	0	0.48	0.06			0.23

Table 3 shows Prevalence of TMJ bony changes according to sex. The Chi-Square test results showed that there was a significant association between sex and condylar erosion (*P* = 0.008). The prevalence of erosion was higher in men. However, sex was not related to any other variables (*p*-value > 0.05).

**Table 3:** Prevalence of TMD disorders for all participants by age groups

Sex	Osteophyte	Erosion	Flattening	Sclerosis	Ely cyst	Condylar hypoplasia	Condylar hyperplasia	Ankylosis
Male	48	39	30	7	0	2	2	10
*N* = 76	-63%	-51%	-39%	-9.20%	0%	-2.60%	-2.60%	-13%
Female	79	40	53	13	2	2	13	15
M = 123	-64%	-32%	-43%	-10.50%	-1.60%	-1.60%	-10.50%	-12%
*P* Value	0.73	0.008	0.61	0.75	0.52	1	1	0.84

Table 4 shows the prevalence of bony changes according to the sex and side (left, right, or both). Of 127 osteophytes, 52 joints (40.94%) were unilateral, while 75 (50.05%) were bilateral. The difference between these two groups was found to be statistically significant. Furthermore, the prevalence of bilateral osteophyte was found to be significantly higher than unilateral osteophyte in both males and females. This suggests that bilateral osteophytes may be a more common occurrence than unilateral osteophyte in both sexes. The table also shows that the prevalence of bony changes, including osteophytes, sclerosis, and flattening was higher on the left side of the TMJ than on the right side in both males and females. This difference was also statistically significant.

**Table 4:** Prevalence of TMD disorders for all participants by sex and side (Right,Left,Both)

Sex	Side	Osteophyte	Erosion	Flattening	Sclerosis	Ely Cyst	Condylar	Condylar	Ankylosis
							Hypoplasia	Hyperplasia	
Male *N* = 76	Right	6	11	5	3	0	0	0	2
		-6.60%	-14.50%	-6.60%	-3.90%				-2.60%
	Left	7	12	10	4	0	2	2	4
		-9.20%	-15.80%	-13.20%	-5.30%		-2.60%	-2.60%	-5.30%
	Both	35	16	15	0	0	0	0	4
		-46.10%	-21.10%	-19.70%					-5.30%
Female *N* = 123	Right	15	21	18	5	2	2	7	8
		-12.20%	-17.10%	-14.60%	-4.10%	-1.60%	-1.60%	-5.70%	-6.50%
	Left	24	8	24	6	0	0	6	3
		-19.50%	-6.50%	-19.50%	-4.90%			-4.90%	-2.40%
	Both	40	11	11	2	0	0	0	4
		-32.50%	-8.90%	-8.90%	-1.60%				-3.30%

Table 5 shows variable evaluation per mean age. Using paired *t*-test,a strong correlation was recorded between mean age and Ely Cyst, Sclerosis,condylar hypoplasia and ankylosis. The prevalence of Ely Cyst increased with age whereas the prevalence of Sclerosis, condylar hypoplasia and ankylosis decreased with age. Ankylosis, Sclerosis and condylar hypoplasia were found to be more predominant in the group age of 10–30, while the prevalence of Ely Cyst was higher in the age group of 50–70.

**Table 5:** Evaluation of bony changes of TMJ per mean age

Variable	Mean Age	Standard Deviation	*P *value
Osteophyte	YES 33.7	13.6	0.74
	NO 34.4	13.02	
Erosion	YES 34.4	14.5	0.66
	N0 33.6	12.6	
Flattening	YES 34.7	12.6	0.51
	N0 33.5	13.93	
sclerosis	Yes	11	0.037
	28.1		
	NO 34.6	13.4	
Ely cyst	YES	0.7	< 0.001
	55.5		
	NO	13.27	
condylar size	33.1		
	hypo 15.5	3.5	< 0.001
	hyper 27.6	11.2	
	Normal 35	13.3	
Ankylosis	Yes	13.8	0.032
	28.68		
	No	13.12	
	34.7		

Table 6 presents the result of the joint space evaluation according to variables and their resulting *P*-values.

**Table 6:** Joint space evaluation according to variables and their resulting *P*-values

Condylar space Right	Variable		Average	SD	*P* Value	*P* Value nonparametric
	osteophyte	yes	2.9369	1.13997	0.191	0.352
		no	3.2448	1.80205		
	Erosion	Yes	2.7295	0.90388	0.04	0.12
		no	3.2624	1.64948		
	sclerosis	Yes	2.2155	1.34019	0.05	0.016
		No	3.1419	1.40306		
	flattening	Yes	3.0774	1.71997	0.812	0.986
		No	3.0289	1.1658		
	Ely cyst	Yes	2.945	0.78489	0.917	0.926
		No	3.0503	1.42727		
	ankylosis	Yes	1.7032	1.84894	< 0,001	0.001
		no	3.2415	1.24138		
		hypo	3.15	0.54705		
	State- condyle	Hyper	2.546	1.27008	0.36	0.557
		normal	3.0887	1.44212		
Condylar space Left	osteophyte	Yes	3.0432	1.21848	0.213	0.308
		No	3.3033	1.717		
	Erosion	Yes	2.8228	1.05143	0.05	0.03
		No	3.3484	1.58862		
	Sclerosis	Yes	1.8175	1.69519	0.001	0.001
		No	3.2848	1.31081		
	Flattening	Yes	3.1469	1.70704	0.941	0.659
		No	3.1317	1.17601		
	Ely cyst	Yes	3.02	0.89095	0.906	0.841
		No	3.1393	1.42511		
	Ankylosis	Yes	1.7196	1.94613	< 0.001	0.001
		No	3.3408	1.2047		
	State- condyle		3.12	0.57515	0.5	0.013
		hypo				
		Hyper	2.0007	1.51888		
		normal	3.2328	1.38764		

The mean joint space of right and left temporomandibular joint was significantly lower in patients with erosion compared to those without erosion (*P* = 0.04). Similarly, there was a significant difference in mean joint space between patients with sclerosis and those without sclerosis (*P* < 0.001). Additionally, the mean joint space of right and left temporomandibular joint with ankylosis was statistically significantly lower than the mean joint space of patients without ankylosis (*P* < 0.001).

Table 7 presents the mean joint space and Standard deviation values for both male and female participants in both sides of the TMJ. A significant difference was found in the mean joint space between male and female participants, as well as between right and left temporomandibular joint (p ≤ 0.05). In other words, the mean joint space of males was statistically significantly higher than the mean joint space of females in both right and left side of TMJ.

**Table 7:** Mean joint space and Standard deviation of male and female in both sides of TMJ

Condylar Space	Sex	Mean joint Space		*P* Value
		Average	SD	
Right	Male	3.4112	1.64687	0.005
	Female	2.8323	1.22179	
Left	Male	3.434	1.67242	0.023
	Female	2.9659	1.21174	

## Discussion

The aim of this study was to evaluate the changes in the temporomandibular area of the jaw in patients with TMD and the relationship between age, sex, and type of change using CBCT.

TMD is a major public health problem that can disturb the health of affected individuals. It has a multifactorial etiology associated with several risk elements that play a major role in the initiation, spread, and exacerbation of TMD symptoms [20]. Identifying these factors is crucial in determining the appropriate treatment [21]. Understanding TMD prevalence, incidence, and other characteristics in populations around the world can help improve diagnostic methods and prevent the development of TMD. The dental practitioners frequently encounter patients with TMDs, and addressing this condition is important for maintaining the overall health and well-being of affected individuals.

CBCT has been recognized as a reliable method for the examination of the osseous components of the TMJ. This technique is easy to perform, is reproducible and delivers a relatively low dose to the patient. Its usefulness has been previously described in the literature [22].

In the present study, various forms of bony changes including osteophyte, erosion, sclerosis, flattening, ankylosis, Ely cyst, hyperplasia and hypoplasia in the condyle were observed. In addition, influence of bony changes on each other was assessed considering *P* value = 0.05. Results showed that presence or absence of radiologic lesion in one side of condyle does not affect the presence or absence of lesion in other side.

In our study, 62% of patients were female and 38% were male. Similarly, many studies have concluded that TMD is more frequent in women than men [18, 22–24]. The greater occurrence in women may be explained by the hormonal influences of estrogen and prolactin, which may exacerbate degradation of cartilage and articular bone in addition to stimulating a series of immunological responses in the TMJ [25, 26]. As a result, Sex is a significant risk factor for TMJ disorders apart from parafunctional habits.

Based on the findings of the findings of the current study, the prevalence of bony changes in the TMJ are as follows:

1-Osteophyte (63.5%) 2-Flattening (42%) 3-Erosion (40%) 4-Ankylosis (12.5%) 5-Sclerosis (10%) 6-condylar Hyperplasia (7.5%) 7-condylar Hypoplasia (2%) and 8-Ely cyst (1%).

This result was in accordance with that of dos Anjos Pontual [18] and contrary to the study of Zhao et al., Bae et al. [14, 19]. With regard to combinations of bone changes, osteophytes accompanied by flattening was more prevalent in our study, differing from the findings by K. S. Nah [9] that found sclerosis as the most prevalent bony change.

According to the present study, the mean age of patients with ankylosis in the condylar head was 28 years old (*P* value = 0.032) which was statistically significantly compared to patients without ankylosis (34 years old). These results are in agreement with the findings of L. B. Kaban et al. [27] and Dongmei He et al. [28]. It suggests that ankylosis of the condylar head is more likely to occur at a younger age, and highlights the importance of early detection and intervention for this condition to prevent further complications and improve outcomes.

The present results showed a significant relationship between age and Ely Cyst. These finding are consistent with previous studies conducted by Sun mee bae et al. studies [19].

Emshof et al. [24] found that 60% of patients with mean age of 37 had erosion. In this study 39% of patients with mean age of 34 had erosion.

Similar to findings of Cömert Kiliç S et al. [29], our study showed no significant relationship between aging and prevalence of degenerative bony changes of TMJ.

In our study, we found that bilaterally occurring osteophyte were significantly more common than unilateral osteophytes in both male and female subjects. However, we did not observe a significant association between bilateral and unilateral occurrence of other studied bony changes. These results are consistent with those reported by A. M. Ferraz et al. [30].

The present results found no significant relationship between age and condylar erosion. This result contrasts with the findings of studies of M. Imanimoghaddam [12], who reported a positive correlation between age and the prevalence of condylar erosion. This disagreement could be attributed to the differences in the age distribution of the study populations, as well as variations in the diagnostic criteria used to assess condylar erosion.

Our study found no statistically significant association between age and osteophyte which is opposite to K. Alexiou et al. [22] and Paknahad et al. [31] findings that concluded the prevalence of osteophyte increases with age. The discrepancy in results may be due to differences in study population. Additionally, genetic and environmental factors may contribute to the development and progression of osteophytes, which could var across different populations and geographic regions.

Our study found a significant negative correlation between age and sclerosis, as evidenced by the statistically significant difference in mean age between patients with and without sclerosis. Specifically, we observed a decrease in the prevalence of sclerosis with increasing age. This result contradicts the findings of K. Alexiou et al. [22] who reported a positive correlation between age and sclerosis. This disagreement could be attributed to the different methodologies, patient population or random variations in the data.

Indeed, further investigations with larger sample sizes are needed to confirm or refute the results of the present investigation. This study has some design and technical limitations. First, since it was a retrospective study, it was difficult to clearly classify complex clinical symptoms. Second, the fluctuating nature of TMD symptoms and signs was not contemplated in the cross-sectional design [32].

It would be beneficial to conduct longitudinal studies to better understand the progression and natural history of TMD signs and symptoms. This would provide valuable insights into the underlying mechanisms and potential treatment options for TMD. Moreover, utilizing magnetic resonance imaging (MRI) would provide detailed information on soft tissue changes in TMD. This could help identify specific structural abnormalities or abnormalities in the TMJ that may be associated with TMD symptoms. Lastly, studying TMD in children would be important as it could provide insights into the early development and progression of TMD. Understanding the factors that contribute to TMD in children could potentially lead to early interventions and prevention strategies.

## Conclusion

Based on the findings of this research, the prevalence of bony changes of TMJ were osteophyte, flattening of the articular surface, erosion, ankylosis, sclerosis, hyperplastic condyles, hypoplastic condyles and Ely Cyst respectively. According to the results of this study, the prevalence of bony changes is more common in women. Sex has been established as a significant risk factor for TMJ disorders apart from parafunctional habits. The prevalence of erosion was higher in men. However, sex was not related to any other variables. CBCT is an accurate 3-dimensional imaging modality for assessment of TMJ bony structures.

## Data Availability

The authors confirm that the data supporting the findings of this study are available within the article and its supplementary materials.
